# Evaluation of prehospital preparedness for major incidents on a national level, with focus on mass casualty incidents

**DOI:** 10.1007/s00068-023-02386-7

**Published:** 2023-12-20

**Authors:** Kristina Stølen Ugelvik, Øyvind Thomassen, Geir Sverre Braut, Thomas Geisner, Janecke Engeberg Sjøvold, Joakim Agri, Carl Montan

**Affiliations:** 1 University of Bergen, Bergen, Norway; 2 Regional Trauma Centre, Haukeland University Hospital, Bergen, Norway; 3 HEMS, Haukeland University Hospital, Bergen, Norway; 4 Norwegian Air Ambulance Foundation, Oslo, Norway; 5 Stavanger University Hospital, Stavanger, Norway; 6 Western Norway University of Applied Sciences, Stavanger, Norway; 7 Gastrosurgical Department, Haukeland University Hospital, Bergen, Norway; 8 Department of Molecular Medicine and Surgery, Karolinska Institutet, Stockholm, Sweden

**Keywords:** Mass Casualty Incidents, Trauma, Major Incidents, Prehospital preparedness, Disaster planning

## Abstract

**Purpose:**

To investigate prehospital preparedness work for Mass Casualty Incidents (MCI) and Major Incidents (MI) in Norway.

**Method:**

A national cross-sectional descriptive study of Norway’s prehospital MI preparedness through a web-based survey. A representative selection of Rescue and Emergency Services were included, excluding Non-Governmental Organisations and military. The survey consisted of 59 questions focused on organisation, planning, education/training, exercises and evaluation.

**Results:**

Totally, 151/157 (96%) respondents answered the survey. The results showed variance regarding contingency planning for MCI/MI, revisions of the plans, use of national triage guidelines, knowledge requirements, as well as haemostatic and tactical first aid skills training. Participation in interdisciplinary on-going life-threatening violence (PLIVO) exercises was high among Ambulance, Police and Fire/Rescue Emergency Services. Simulations of terrorist attacks or disasters with multiple injured the last five years were reported by 21/151 (14%) on a regional level and 74/151 (48%) on a local level. Evaluation routines after MCI/MI events were reported by half of the respondents (75/151) and 70/149 (47%) described a dedicated function to perform such evaluation.

**Conclusion:**

The study indicates considerable variance and gaps among Prehospital Rescue and Emergency Services in Norway regarding MCI/MI preparedness work, calling for national benchmarks, minimum requirements, follow-up routines of the organisations and future reassessments. Implementation of mandatory PLIVO exercises seems to have contributed to interdisciplinary exercises between Fire/Rescue, Police and Ambulance Emergency Service. Repeated standardised surveys can be a useful tool to assess and follow-up the MI preparedness work among Prehospital Rescue and Emergency Services at a national, regional and local level.

**Supplementary Information:**

The online version contains supplementary material available at 10.1007/s00068-023-02386-7.

## Introduction

Major incidents (MI) are defined as situations where the available resources are insufficient for the immediate need [1]. Armed conflicts, terrorist attacks, increased travelling, urbanisation, increased population and climate change contribute to the fact that MIs are increasing globally [1, 2]. A holistic approach to preparedness and disaster planning is needed to handle MIs as they are complex and unpredictable events with a high degree of diversity in magnitude and scenarios, causing organisational challenges [3]. MI preparedness requires planning, training, exercise, evaluation and revision [4–10]. A functional trauma system is crucial in managing MI and Mass Casualty Incidents (MCI) [11, 12]. Different tools for evaluation and improvement of the response and preparedness to MI are found in the literature; local visits, interviews, surveys, retrospective reports, surge capacity tests and reviewed exercises are described methods [12–18]. MI organisation varies between countries due to geographical, legal, organisational, cultural, and resource differences. These factors must be considered when investigating and evaluating MI preparedness [10, 15, 16, 19].

The Norwegian preparedness system is based on a civilian system and the defence sector [20]. The Ministry of Health carries the responsibility for coordination and preparedness in the health sector, and the Directorate of Health holds the role as adviser, policy implementer and administrator of statutes/regulations [20]. Four Regional Health Authorities provide Specialist Health Care through the Health Trusts. Specialist services include hospitals, patient transportation (including Helicopter Emergency Services (HEMS)) and the Norwegian Emergency Medical Communication Central (EMCC). Municipalities are responsible for Primary Health Care (PHC) and provide General Practitioners on call through municipal or inter-municipal Emergency Call Centrals [21]. A revised national trauma plan was implemented in 2017 with the aim to reduce differences between regions, define quality of care indicators and describe competence requirements [22].

The Rescue and Emergency Services represent the first part of the trauma chain. The cooperation principle is crucial [20, 23]. For background information regarding the Norwegian Prehospital Services see Online Resource 1. Two Joint Rescue Centrals (JRC) are responsible for the coordination of operations at sea disposing Search and Rescue Helicopter Services (SAR). The Joint Rescue Centrals assist Local Rescue Centrals (LRC), which coordinate Rescue operations on land [24, 25]. Transport can be a challenging task in Norway due to the northern latitude, long coastline and harsh weather. Anaesthetic doctors are members of the HEMS and SAR crew, contributing with triage, treatment and transport. Criteria for activating the trauma team and a distribution key for trauma patients are described in a national trauma plan [22]. In 2020, a revised national guide for prehospital mass-casualty triage was provided based on the Sort, Assess, Lifesaving Interventions, Treatment/Transport (SALT) principle [26, 27].

National guidelines are provided for organisation of the Rescue Service [24] to clarify roles, tasks and command lines on scene between Medical Incident Commander (MIC), Police Incident Commander (PIC) and Rescue Incident Commander (RIC) and between the General Practitioner on call and the HEMS/SAR doctor [25, 28]. After the twin attacks in Oslo in 2011, a national guideline for situations with on-going life-threatening violence (PLIVO) was implemented to guide the cooperation among Emergency Services [29], and a National Digital Emergency Communication Network (“Nødnett”) was established for communication between the Rescue and Emergency Services [30].

Studies have shown that experience and competency among Prehospital Health providers have relevance for optimal management of MCI/MI [31, 32]. The experience with MCI/MI among Prehospital Rescue and Emergency providers in Norway is limited due to a peaceful context and a generally low trauma burden [15, 21]. The lack of exposure to MI, highlights the need for training and drills/exercises [10, 33]. According to the National Health Preparedness Plan “*All entities in the health and care sector are responsible for ensuring their own personnel and organisation are instructed and trained”* [20]. The responsibility to plan and implement an annual national health drill is given to the Directorate of Health. No directives are found on a regional level regarding exercises/drills.

There is no national method for following up the prehospital contingency work for MCI/MI in Norway. The primary aim of this study was to describe the current medical preparedness for MI among Norwegian Prehospital Rescue and Emergency Services using an adapted national standardised survey based on the methodology used by Agri et al. in a Swedish study [19]. Secondary aims were to analyse the findings according to the existing national guidelines and to evaluate the relevance of the methodology.

### Method

**Fig. 1:**
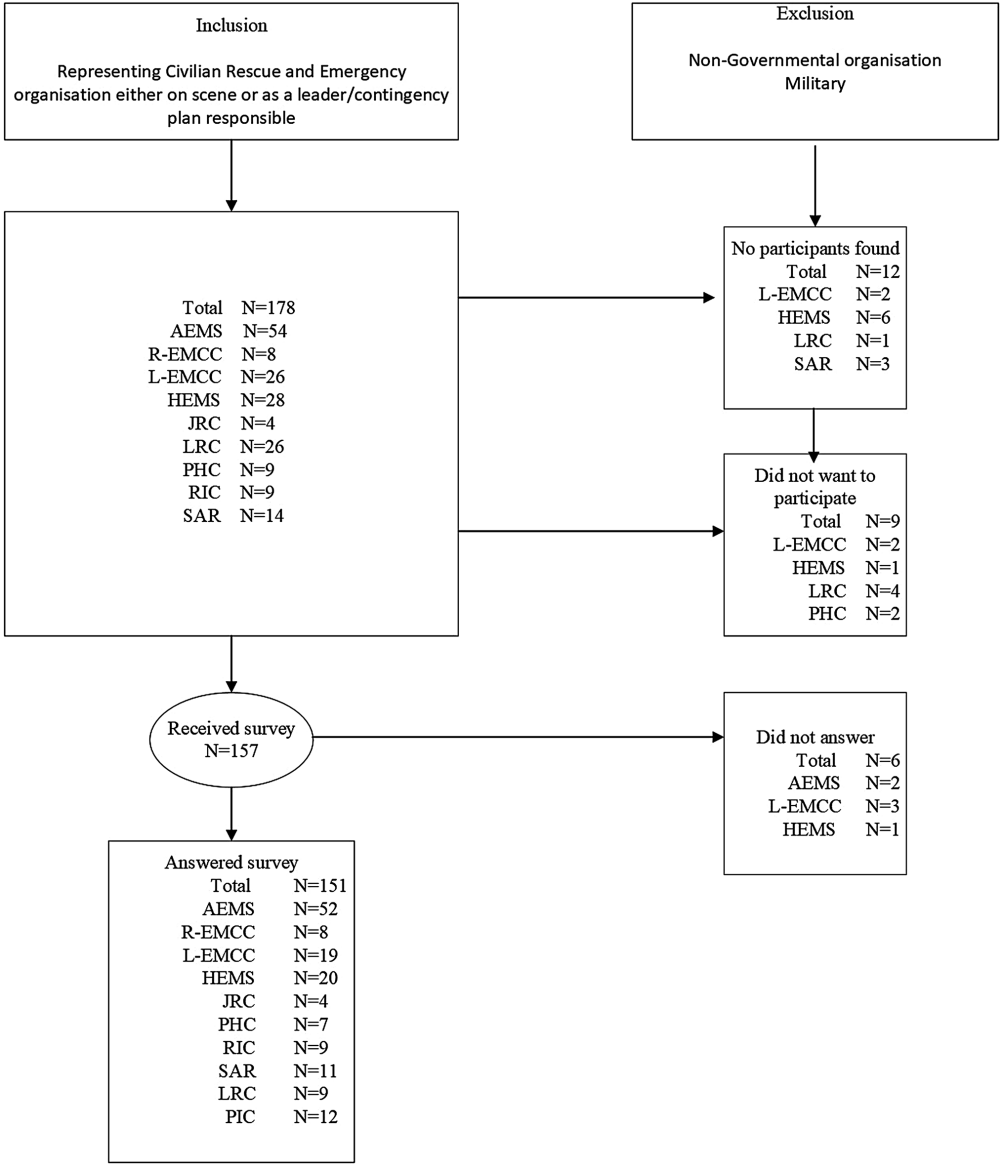
*AEMS* ambulance emergency services, *HEMS* helicopter emergency services, *L-EMCC*  local emergency medical communication central, *R-EMCC*  regional emergency medical communication central, *SAR* Search and Rescue Helicopter Services, *JRC*  joint rescue central, PHC primary health care general practitioner on call, *RIC*  rescue incident commander, *PIC* police incident commander, *MIC* medical incident commander, *LRC* local rescue central

A national cross-sectional descriptive study of Norway’s prehospital MI preparedness was conducted with the following focus areas; organisation, planning, training, exercise/simulation and evaluation. A secure and well adapted software was utilised to collect web-based data from a representative selection of respondents from Prehospital Rescue and Emergency providers (Table 1 and Fig. 1). Owing to the extensive cooperation between the civilian Emergency Services on scene, relevant organisations outside the health sector were invited to participate in the study. The organisations included, and the functions and number of respondents invited to the survey is described in Table 1. Background information regarding the included organisations, their role in MCI/MI events and the cooperation principle is available as a supplementary file (Online Resources 1).

**Table 1:** Included Rescue and Emergency Organisations, abbreviations and survey respondents’ function in Mass Casualty Incidents

Organisation invited (Number included/number existing on a national level)	Abbreviation	Function of respondent(s) invited to answer the survey	Respondents invited to participate
Joint Rescue Centrals (2/2 included)	JRC	-2 Operational leaders per central; receive alarms, coordinate rescue operations at sea/coastline. Coordinates SAR	4
Local Rescue Centrals (12/12 Police Districts + Svalbard included)	LRC	-1 Operational leader or contingency plan responsible for alarms on land	13
Emergency Medical Communication Centrals (4/4 Regional, 13/13 Local included)	EMCC	-1 Operational leader. Receive alarms and coordinate patient transport resources -1 Leader or contingency plan responsible	34
Police Incident Commander (12/12 Police Districts + Svalbard included)	PIC	-1 Operational leader police on scene	13
Rescue Incident Commander (All NORCE “Watchtower” Municipalities included)	RIC	-1 Operational leader Rescue and Fire on scene municipal/inter-municipal _*1*_	9
Primary Health Care Emergency Central (All NORCE “Watchtower” Municipalities included)	PHC	-1 General Practitioner on call sent to the scene. Can function as Medical Leader Health (MLH) _*2*_	9
Ambulance Emergency Medical Services (18/18 Health Trusts included)	AEMS	-1 Medical Incident Commander (MIC) District -1 Medical Incident Commander (MIC) Central areas -1 Leader with contingency plan responsibility	54
Helicopter Emergency Medical Services. Regional EMCC coordination (14/14 Bases included)	HEMS	- 1 anaesthetic doctor. Medical treatment, triage, patient transport. Can function as Medical Leader Health (MLH) on scene - 1 Leader or contingency plan responsible	28
Search and Rescue Helicopter Services. JRC coordination (7/7 Bases included)	SAR	-1 anaesthetic doctor. Medical treatment, triage, patient transport. Can function as Medical Leader Health (MLH) on scene - 1 Leader or contingency plan responsible	14

^1^Elverum municipality represented with RIC from elverum as well as RIC from Hedmark intermunicipal fire and rescue central consisting of 11 municipalities including Elverum.

^2^Austevoll municipality represented by general practitioner on call from Austevoll and by Bjørnafjorden intermunicipal call central which includes Austevoll

As health preparedness was the focus, more respondents were included from the Health Sector. The Specialist Health Care was prioritised, managing the most severely injured patients more likely to be affected by a less optimal level of care during MI. The NORCE “Watchtower Municipalities”, representing the Norwegian General Practitioner on call services, were selected as representatives for Municipality-based Emergency and Rescue Services (Table 1 and Fig. 1) [34]. Non-Governmental Organisations and the military were excluded from the survey.

The survey consisted of 59 questions, including multiple-choice and free-text options (appendix number 1). The survey design and focus areas were based on the survey conducted by Agri et al. on Swedish prehospital MI preparedness [19]. Questions were adapted to Norwegian legislation and guidelines. The following categories were included in the survey:

- Background information from the respondents;
- Organisation; triage system, tasks, communication;
- Contingency plans and action cards;
- Education and training;
- Exercises and simulations;
- Evaluation;
- Areas of improvement.

An email with information about the study was sent to leaders of the organisations one week before the survey was rolled out. Leaders chose respondents to represent their organisation. As some respondents worked in several prehospital organisations, we asked them to answer on behalf of the organisation, as specified in the letter of invitation. Discussion with colleagues and information search was encouraged. For those who had several roles in their organisation, their highest-ranking position was asked for when answering the survey. The participants received a phone call before receiving the survey via email. The data were collected in the period 24.10.2022 -17.11.2022. Those that did not answer were reminded by phone call and email. Some organisations or participants did not want to participate in the study, others were missed due to lack of names or contact information and some did not answer despite follow up (Fig. 1).

Analysis of quantitative data was performed by exporting data to IBM SPSS Statistics for Windows, Version 26.0. Armonk, NY: IBM Corp and Microsoft Corporation Microsoft Office 365 Excel Version 16.0 One Microsoft Way, Redmond, WA 98052-6399. Regarding answers to multiple-choice questions, the different Rescue and Emergency Services were compiled, and the percentage distribution was calculated and visualised in tables. Median and IQR was used for descriptive statistics.

## Results

Answers were collected from 151/157 (96%) of the respondents receiving the survey (Fig. 1). All Norwegian Health Trusts with prehospital functions were represented by a minimum of 3 (3–18) respondents, varying as EMCC/HEMSservices were not found in all Health Trusts. The Police was represented by 11 out of 13 Police Districts (85%). Among the respondents, 39/151 (26%) had either leader responsibility or were responsible for the MCI/MI contingency plan in their organisation. Half of the responding Medical Incident Commanders represented rural attachment areas (17/33).

### Organisation, communication and triage

A majority of the respondents stated that the Medical Incident Commander was either the most experienced ambulance worker on the first arriving ambulance on scene (94/150 (63%)) or the Operational Leader of the Ambulance Emergency Services (47/150 (31%)) where the role existed. Accordingly, 131/150 (87%)) pointed to the Medical Incident Commander and 8/150 (5%)) the Operational Leader as responsible for communication and cooperation with the Police Incident Commander and the Rescue Incident Commander on scene. Regarding health resource management on scene, 100/148 (68%) of the respondents stated the Medical Incident Commander and 8/149 (5%) the Operational Leader as responsible.

Most of the respondents recognised the Medical Leader Health position to be held by a medical doctor, as either HEMS (54/150 (37%)) or General Practitioner on call (70/150 (47%)) was chosen. For medical decisions on scene, the majority stated that this was a medical doctor’s responsibility; 100/148 (68%) answered the Medical Leader Health and 14/148 (9%) the General Practitioner on call. Others (36/148 (24%)) considered medical decisions a joint responsibility between the Medical Incident Commander and the Medical Leader Health. According to 72/150 (48%) of the respondents, patient distribution was a joint responsibility between the Medical Incident Commander and the Medical Leader Health while 64/150 (43%) answered the Medical Leader Health or the General Practitioner.

Answers regarding the decision whether first aid could be performed on scene varied, as some stated the Police Incident Commander 93/151 (62%), some a joint decision between 2 or more Emergency Services 26/151 (17%) and others the Medical Incident Commander 17/151 (11%). Guidelines for Health Personnel regarding “how to act when arriving first on scene during a possible terrorist attack” were found in the contingency plans of 41/89 (46%) of respondents from Ambulance Emergency Medical Services, Helicopter Emergency Medical Services, Search and Rescue Helicopter Services and General Practitioner on call.

SALT was identified as the primary triage tool for MCI in the organisations of 77/150 (51%) of the respondents, with variance among Rescue and Emergency Services (Table 2). Several MCI/MI primary report forms were used; HENSPE (34/150 (23%)), METAFOR (12/150 (8%) and METHANE 9/150 (6%). Of the Police respondents, 11/21 (52%) stated that a national reporting form was utilised in MCI/MI.

**Table 2:** Prehospital Rescue and Emergency Services reported use of National triage guidelines for Mass Casualty (SALT) in their organisation

	AEMS	HEMS	SAR	PHC	RIC	PIC
SALT primary triage guideline use	37/51 (73%)	15/20 (75%)	7/11 (63%)	3/ 7 (42%)	1/9 (11%)	2/12 (17%)

*AEMS* ambulance emergency medical services, *HEM* helicopter emergency medical services, *SAR*  search and rescue helicopter services, *PHC* primary health care general practitioner on call, *RIC* rescue incident commander, *PIC* police incident commander

### MCI/MI contingency plans

Most respondents 131/151 (87%) stated that they had a local contingency plan for MCI/MI in their organisation. On a regional level, 98/134 (65%) replied that they had a contingency plan for MCI/MI. The local contingency plan was adapted to the regional contingency plan for MCI/MI in 64/98 (64%) of the organisations. Variance in the contingency plan work was detected among Emergency Services (Table 3).

**Table 3:** Contingency plans for MCI/MI among Rescue and Emergency Services

	Local contingency plan for MCI/MI	Regional contingency plan for MCI/MI	Local contingency plan for MCI/MI adjusted to regional contingency plan	Revision of contingency plan MCI/MI < 12 months	Activation of contingency plan MCI/MI < 24 months
AEMS	48/52 (93%)	32/52 (62%)	18/32(56%)	11/52 (21%)	14/52 (26%)
HEMS	20/20 (100%)	16/20 (80%)	7/16 (35%)	9/20 (45%)	10/20 (50%)
SAR	11/11 (100%)	9/11 (82%)	7/9 (78%)	5/11 (46%)	7/11 (64%)
PHC	5/7 (72%)	2/7 (29%)	2/2 (100%)	3/7 (43%)	1/7 (14%)
RIC	4/9 (44%)	1/9 (11%)	1/1 (100%)	1/9 (11%)	2/9 (22%)
PIC	11/12 (92%)	10/12 (83%)	7/10 (70%)	7/12 (58%)	7/12 (58%)
JRC	1/4 (25%)	1/4 (25%)	1/1 (100%)	0/4 (0%)	2/4 (50%)
R-EMCC	8/8 (100%)	6/8 (75%)	4/6 (67%)	5/8 (63%)	6/8 (75%)
L-EMCC	18/19 (94%)	15/19 (78%)	12/15 (80%)	8/19 (42%)	7/19 (37%)
LRC	7/9 (78%)	6/9 (67%)	5/6 (83%)	6/9 (67%)	4/9 (44%)

*MCI*  mass casualty incidents, *MI*  major incidents, *AEMS*  ambulance emergency medical services, *HEMS* helicopter ambulance emergency medical services, *SAR*  search and rescue helicopter services, *PHC*  primary health care general practitioner on call, *RIC* rescue incident commander, *PIC* police incident commander, *JRC*  joint rescue central, *R/L EMCC*  regional/local emergency medical communication central, *LRC*  local rescue central

Concerning contingency plan revision, 72/149 (48%) answered that their contingency plan for MCI/MI was revised either yearly or revised within the last year. However, 47/149 (32%) “did not know” and 30/149 (20%) replied that the last revision was more than 2 years ago. Regarding follow up of MCI/MI contingency plan knowledge among staff, 57/150 (38%) answered that all new employees must read the contingency plan, 24/150 (16%) stated that the individual employee decided whether to read the contingency plan, 17/150 (11%) replied that no guidelines existed within their organisation regarding the contingency plan and 17/150 (11%) “did not know”. Knowledge about the MCI/MI contingency plan in their organisation was based on trust according to 49/150 (33%) of our respondents, 27/150 (18%) “did not know” how knowledge about the contingency plan for MCI/MI was followed up in their organisation and 11/150 (7%) reported yearly follow up. Web-based follow up was used in the organisation of 16/150 (11%) of the respondents and 14/150 (9%) answered that review was performed in plenary. Regarding action cards, 80/150 (53%) answered that some of the functions in the contingency plan had action cards whereas 48/150 (32%) stated that all functions had action cards.

The time since the last activation of the contingency plan for MCI/MI varied among respondents, as 59/149 (40%) replied that the MCI/MI contingency plan had been activated within the last two years and 14/149 (9%) replied that the plan was never activated. The time since the last activation of the MCI/MI contingency plan was found to vary among Prehospital Rescue and Emergency Services (Table 3).

Chemical, Biological, Radiological, Nuclear, Explosive (CBRNE) incidents (122/149 (82%)) and MCI (110/149 (74%)) were recognised as the most frequent scenarios described in the contingency plans (Table 4).

**Table 4:** Scenarios described in the contingency plans in the respondents` organisation: multiple answer question

Scenarios described in the contingency plan for MCI/MI	Number (%)
CBRNE	122/149 (82%)
Mass Casualty Incidents	110/149 (74%)
Fire	93/149 (62%)
PLIVO	93/149 (62%)
Epidemic	90/149 (60%)
Network breakdown	84/149 (56%)
Natural disasters	76/149 (52%)
Evacuation of buildings	66/149 (44%)
Security threat	61/149 (41%)
Change in infrastructure	55/149 (37%)
Terrorist attack	54/149 (36%)
Lack of water	43/149 (29%)
Hypothermia	36/149 (24%)
Others	36/149 (24%)
Inhalation injury	24/149 (16%)
Psychological trauma	24/149 (16%)
Did not know	13/149 (9%)
No scenarios	7/149 (5%)

*mci* mass casualty incident, *MI * major incidents, *CBRNE*  chemical, biological, radiological, nuclear, explosive incidents, *PLIVO*  Shortening for on-going life-threatening violence

### Training and communication

Regarding training for MCI/MI, 68/150 (45%) of the respondents reported that training of staff to improve communication was conducted. An almost equal number 67/150 (45%) of respondents did not train communication in their organisations. Communication training practices varied among Prehospital Rescue and Emergency Services (Table 5). In a case of breakdown of the National Digital Network “Nødnett”, plans and tests for alternative tools of communication were performed in the organisation of 73/150 (49%) of the respondents, 49/150 (32%) replied that no alternative communication tools had been tested and 28/150 (19%) answered “did not know”. Variance in “Nødnett” breakdown preparedness among Rescue and Emergency Services was detected (Table 5).

**Table 5:** Communication training for MCI/MI and training or alternative plans for “Nødnett” breakdown among Rescue and Emergency Services:

	Communication training for MCI/MI in the organisation	“Loss of nødnett” alternative plan or exercise in the organisation
AEMS	28/52 (54%)	22/52 (42%)
HEMS	6/30 (20%)	9/30 (30%)
SAR	3/11 (27%)	3/11 (27%)
PHC	5/7 (71%)	1/7 (14%)
RIC	1/3 (33%)	5/9 (56%)
PIC	6/12 (50%)	6/12 (50%)
JRC	1/4 (25%)	3/4 (75%)
R-EMCC	0/8 (0%)	5/8 (63%)
L-EMCC	10/19 (53%)	12/19 (63%)
LRC	6/9 (67%)	7/9 (78%)

*MCI*  mass casualty incident, *MI*  major incident, *AEMS*  ambulance emergency medical services, *HEMS *  helicopter ambulance emergency medical services, *SAR*  search and rescue helicopter services, *PHC*  primary health care general practitioner on call, *RIC*  rescue incident Commander, *PIC* Police Incident Commander, *JRC*  joint rescue Central, *R/L EMCC*  regional/local emergency medical Communication Central, *LRC*  local rescue central

Tactical Emergency Medical course/training was provided to one or more employees in the organisations of 20/150 (13%) of the respondents. Variance among different Prehospital Emergency Services was found (Table 6). However, 104/150 (69%) stated that one or more employees in their organisation had received “haemostatic skills” training, with variance among Rescue and Emergency Services (Table 6). According to answers, most AEMS and SAR/HEMS transport units carried 2–3 tourniquets. Police answered that each transport unit most often carried 4–5 tourniquets, in contrast to 1 tourniquet per transport unit reported by Rescue Incident Commanders and General Practitioners on call.

**Table 6:** Haemorrhagic skill training and tactical first aid training offered to one or more staff in Rescue and Emergency Services represented on scene

	AEMS	HEMS	SAR	PHC	RIC	PIC
Haemorrhagic training	39/52 (75%)	19/20 (95%)	9/11 (82%)	1/7 (14%)	6/9 (67%)	12/12 (100%)
Tactical first aid training	5/52 (10%)	4/20 (20%)	1/11 (9%)	1/7 (14%)	1/9 (11%)	5/12 (42%)

*AEMS*  ambulance emergency medical services, *HEMS*  helicopter ambulance emergency medical services, *SAR* search and rESCUE helicopter services, *PHC*  primary health care general practitioner on call, *RIC* rescue incident commander, PIC = police incident Commander

### Exercises/simulations

Concerning Cooperation and Leadership exercises with other prehospital organisations within the last year, 90/150 (61%) of respondents’ organisations had participated. In comparison, 61/150 (40%) answered that internal Cooperation and Leadership exercises (without other organisations) took place. Within the last year 81/150 (54%) of the respondents had participated in Cooperation and Leadership exercises. Ambulance Emergency Medical Services, Fire/Rescue and Police were the most frequently reported participants (Table 7). Considerable variance was detected between different Rescue and Emergency Services regarding participation in Leadership and Cooperation exercises and patient-case simulations (Table 8).

**Table 7:** Participating organisations in Leadership and Cooperation exercises as stated by respondents. Multiple answer question

Rescue and Emergency Service	Rescue and Emergency Services reported with participation in Leadership and Cooperation exercises by respondents (Multiple answer question)
AEMS	124/151 (82%)
Rescue and fire	124/151 (82%)
Police	123/151 (81%)
EMCC	115/151 (76%)
HCG/Hospitals	77/151 (51%)
HEMS	63/151 (42%)
PHC	60/151 (40%)
PHC Emergency Call Central	40/151 (27%)
JRC	27/151 (18%)
Others	19/151 (13%)
«Did not know»	13/151 (9%)

*AEMS*  ambulance emergency medical services, *EMCC* emergency medical communication central, *HCG *hospital command group, *HEMS* helicopter emergency medical services, *PHC* primary health care general practitioner on call, *JRC*  joint rescue central

**Table 8:** Leaderships and Coordination exercise and patient-case simulation activities among prehospital rescue and emergency services last 12 months

	Respondents’ participation in Leadership and Cooperation exercises < 12 months	Organisations participated in Leadership and Cooperation exercise with other prehospital services involving hospital < 12 months	Organisations participated in internal Leadership and Cooperation exercise without other organisations < 12 months	Organisations participation in patient-case simulation < 12 months
AEMS	27/51 (53%)	30/51 (59%)	22/51 (43%)	40/51 (78%)
HEMS	7/20 (35%)	14/20 (70%)	5/20 (25%)	16/20 (80%)
SAR	4/11 (36%)	6/11 (55%)	5/11 (45%)	8/11 (73%)
PHC	1/7 (14%)	3/7 (43%)	1/7 (14%)	2/7 (29%)
RIC	6/9 (67%)	5/9 (56%)	6/9 (67%)	3/9 (33%)
PIC	10/12 (83%)	8/12 (67%)	7/12 (58%)	11/12 (92%)
JRC	2/4 (50%)	3/4 (75%)	1/4 (25%)	0/4 (0%)
R-EMCC	4/8 (50%)	4/8 (50%)	2/8 (25%)	3/8 (38%)
L-EMCC	8/19 (42%)	11/19 (58%)	4/19 (21%)	13/19 (68%)
LRC	8/9 (89%)	6/9 (67%)	8/9 (89%)	6/9 (67%)

*AEMS* ambulance emergency medical services, *HEMS* helicopter ambulance emergency medical services, *SAR*  search and rescue helicopter services, *PHC* primary health caregeneral practitioner on call, *RIC*  rescue incident commander, *PIC*  police incident commander, *JRC*  joint rescue central, *R/L EMCC* regional/local emergency medical communication central, *LRC* local rescue central

Patient-case simulations were conducted within the last year by 82/150 (68%) of the respondents’ organisations. A variety of different scenarios were used in patient-case simulations. PLIVO was the most frequently reported case scenario in the multiple answer question (PLIVO with firearm (119/151 (79%)) and PLIVO without firearm (96/151)). Regarding simulations of terrorist attacks or disasters with multiple injured, 21/151 (14%) reported that their organisations participated in one or more simulation within the last 5 years on a regional level and 74/151 (48%) on a local level.

### Evaluation

Half of the respondents (75/151) stated having routines in their organisation for a structured MCI/MI follow-up after MI/MCI events. Based on our findings, there was variance in evaluation routines for MCI/MI (Table 9). Among the respondents, 70/149 (47%) worked in an organisation with a dedicated function to evaluate larger events. According to 45/149 (30%) of the respondents no structured follow-up took place in their organisations after MCI/MI events and 30/149 (20%) “did not know”.

**Table 9:** Follow-up and evaluation after MCI/MI events and responsible function for the evaluation

	MCI/MI evaluation	Evaluation responsible function in the organisation
AEMS	27/52 (52%)	17/51 (33%)
HEMS	8/20 (40%)	11/20 (55%)
SAR	5/11 (46%)	6/11 (54%)
PHC	3/7 (43%)	1/7 (14%)
RIC	3/9 (33%)	4/9 (44%)
PIC	6/12 (59%)	10/12 (83%)
JRC	1/4 (25%)	2/4 (50%)
R-EMCC	5/8 (63%)	5/8 (63%)
L-EMCC	12/19 (63%)	8/18 (44%)
LRC	6/9 (67%)	6/9 (67%)

*MCI* mass casualty incident, *MI*  major incident, *AEMS* ambulance emergency medical services, *HEMS* helicopter ambulance emergency medical services, *SAR* search and rescue helicopter services, *PHC* primary health care general practitioner on call, *RIC* rescue incident commander, *PIC* police incident commander, *JRC* joint rescue Central, *R/L EMCC* regional/local emergency medical communication Central, *LRC*  local rescue central

### Participants’ opinions regarding improvement areas

In the final part of the survey, concerning suggestions for MI improvement, participants graded the importance of several focus areas in MI preparedness. Unequivocal leadership, improved cooperation between Prehospital Rescue and Emergency Services and exercises/simulations were the highest rated (Table 10).

**Table 10:** Respondents grading areas of MI improvement from 0 to 10 (0 = No importance, 10 = Great importance)

Improvement area	Median	Min	Max	IQR
Unequivocal Leadership	10,0	4	10	1
Improved cooperation btw Emergency and Rescue Services	10,0	5	10	2
Exercises/simulations	10,0	3	10	2
Improved communication	9,0	5	10	2
Improved civilian and military cooperation	8,5	2	10	3
International knowledge exchange	8,0	1	10	3
Scientific Research	8,0	1	10	3
Emergency storage	8,0	0	10	3

## Discussion

A survey may be used as an assessment tool to evaluate MI preparedness work on a national level. Based on this study there seems to be several areas of improvement in the Norwegian Rescue and Emergency Services regarding preparedness for MCI/MI. The results suggest variance in the preparedness work among Rescue and Emergency Services and variable adherence to the existing national guidelines.

In Norway, the responsibility for preparedness work for MCI/MI is to a large degree placed locally upon the individual organisation in the trauma chain, unlike for example in Israel where there is a national master MCI plan with annual inspections of the hospitals, and where the Ministry of Health conduct full-scale drills at the hospitals biennially [35]. Although the study describes the Norwegian prehospital MCI/MI preparedness work, our findings have a general value likely to be relevant to other countries. Despite the differences in the structure, organisation and command lines in the prehospital MCI/MI preparedness system, the use of a web-based survey was found useful as an assessment tool to evaluate preparedness work on a national level both in Norway and Sweden [19]. A survey may be adapted to assess different focus areas, organisations, management levels and contexts. Implementation of national benchmarks and minimal requirements, followed up by regular evaluation by national authorities with the use of a web-based survey, could possibly reduce the variation in preparedness work identified and improve the quality of care. The current geopolitical situation and climate change scenarios prove this topic to be of importance for further studies outside the Nordic context.

### Organisation

The role of the Medical Leader Health and the Medical Incident Commander on scene as described in the national guidelines could be further clarified in interdisciplinary training and exercises to avoid possible confusion among other Rescue and Emergency Services [28]. The national guidelines consider each event as unique and encourage a dynamic structure. Lack of specification in the guidelines regarding the Medical Leader Health competency, specifically the General Practitioner versus the Specialist in Anaesthesiology, could result in fragmentation of responsibility between Primary Health Care and Specialist Health Care. Uncertainty regarding the qualification requirements of the Medical Leader Health role could affect commitment and participation in interdisciplinary exercises. Our survey suggests that the national triage guidelines for MCI need to be acknowledged and utilised. For primary report from the scene of an MCI/MI event, several report forms were used. A national standardisation could make the first MCI/MI report from the scene more structured and recognisable across Rescue and Emergency Services.

### Contingency plans

Contingency plans for MCI/MI both on a local and regional level was identified as an area of improvement. Considerable variance was detected among Rescue and Emergency Services regarding the existence of plans and revision routines. Regional contingency plans for MCI/MI were more seldom reported, possibly reflecting the structure of the Norwegian system wherein the individual organisations are given responsibility of disaster planning, training of staff and exercises on a local level [20]. This differs from Sweden, who has a regionally founded system for medical disaster preparedness with Regional Disaster Coordinators on call [19]. Our study indicates that the staff’s knowledge about the organisations` MCI/MI contingency plans to a large extent was based on trust, and many did not have routines for follow-up, corresponding with the results from the Swedish study of prehospital MI preparedness [19].

### Training and communication

Tactical first aid skills could be strengthened among the Emergency providers. Haemorrhagic control skills was identified as an area of improvement in our study corresponding with other studies regarding Norwegian prehospital MCI/MI preparedness [36, 37]. International studies have found that early prehospital external haemorrhage control can reduce morbidity and mortality [38–40]. A national minimum requirement of haemorrhagic skills training for Rescue and Emergency personnel on scene could improve the preparedness for MI. The study suggests that the Rescue and Emergency Services need to plan for and test alternative ways of communication. Challenges with the digital emergency network was experienced both during the Turøy helicopter accident and extreme weather Tor in 2016 [41, 42]. The importance of communication and functioning communication tools in MI is well established [43–45].

### Exercises

Only 59/149 (40%) of the respondents answered that their organisations had activated the MCI/MI contingency plan within the last 2 years, identifying a demand for exercises to ensure that staff are prepared for such events. Our study shows that only 81/150 (54%) of the respondents had participated in Leader and Coordination exercises and 82/150 (68%) of the respondent’s organisations had participated in patient-case simulations within the last year. In line with the study conducted by Blix et al. of the Ambulance Emergency Medical Services in Norway, our study indicates adherence to the mandatory annual PLIVO exercise among some of the Rescue and Emergency Services [36]. However, results suggests that other Emergency providers less frequently participate in Cooperation and Leadership exercises and patient-case simulations. Our findings are corresponding with the cross-sectional study of the Norwegian Helicopter Emergency Medical Services In Major Incidents by Johnsen et al., highlighting limited exposure and a call for interdisciplinary training [33] and the study by Hjortland et al., where only 28% of the Norwegian General Practitioners had participated in an annual interdisciplinary exercise with other Emergency Services [46].

In the study, interdisciplinary Leadership and Coordination exercises with other organisations occurred more frequently than smaller exercises within their own organisation, which should be easier to plan and conduct. The lack of national mandatory exercise minimum standards for other exercises than PLIVO might possibly explain this finding. The use of exercises and tabletop simulations as part of MI preparedness is well established and the benefits of previous exposure to MI exercises and tabletop simulations have been identified in studies from both UK and France after terrorist attacks [5, 6, 47, 48].

### Evaluation

As stated in the national preparedness plan, evaluation after exercises and events should be performed and result in adjustments when needed [20]. Although the study showed variation among Rescue and Emergency Services, the follow up and evaluation routines for MCI/MI were identified as an important area of improvement. Only half of the respondents reported structured follow up after MCI/MI events and less than half had a function responsible for evaluation in their organisation. Useful information about bottlenecks and system errors were identified after terrorist attacks both in Norway and in Europe [8, 43, 44, 47–49]. Having a structured evaluation reporting system after MI can be useful, not only on a local level. Lessons learned after larger events could potentially be used internationally for MI preparedness organising, planning and training. This is an interesting area where further research is required [50, 51].

## Limitations

The study was dependent on the respondents’ own perception of their organisation. Some of the respondents also had multiple roles making their responses possibly biased. Respondents with leadership responsibility might have been less inclined to review weaknesses within their own organisation. Although the study was designed on a tested Swedish model, the validity outside of Norway and Scandinavia could be questioned. Compared to the initial Swedish study using the methodology, the current study had an exceptionally good response rate and good national coverage with all Health Trusts for Ambulance Services represented which likely adds to the validity. The high response rate was not only reflected through close follow up of the respondents by the research team, but also in Prehospital Rescue and Emergency Services with a high degree of cooperation will and a genuine wish to improve the MI system and interdisciplinary MI knowledge.

To highlight the importance of the prehospital cooperation principle, non-health professionals were included in the study, however, some of the questions might not have been relevant for the non-health personnel. The scarce inclusion of representatives from General Practitioners on call and the Fire/Rescue Services could result in an inconsistent representation of the Municipality based Emergency Services. Exclusion of the military and Non-Governmental Organisations, imposes limitations regarding civilian-military cooperation and lack of information regarding the Non-Governmental Organisations` role in Search and Rescue operations.

Sensitive/confidential information gathered in a national survey on preparedness work could represent a security threat, potentially exposing information on individual organisations or on a regional level regarding deficiencies/gaps.

## Conclusion

Considerable variance among Norwegian Prehospital Rescue and Emergency Services regarding MCI/MI preparedness work was observed, indicating a need for national benchmarks and minimum requirements. The national triage guidelines should be acknowledged. Improved communication skills for MCI/MI and improved haemorrhagic and medical tactical first aid skills are called for. Implementation of PLIVO exercises seems to have contributed to interdisciplinary exercises between Fire/Rescue, Police and Ambulance Emergency Medical Services. Other Rescue and Emergency Services as General Practitioner on call/Helicopter Emergency Medical Services/Search and Rescue Helicopter Services could be more involved. Establishing revision routines for MCI/MI contingency plans and follow-up routines regarding contingency plan knowledge among staff were detected as areas of improvement. Further, the need for an evaluation system after MCI/MI was identified.

With adoption to national contexts, a well prepared and structured survey may be used as a tool to help evaluate MI preparedness work on a national level. Together with exercises and surge capacity tests the use of this methodology could be deployed as a national quality assurance tool for minimum requirements, used to detect improvement areas on a regular basis. If correctly implemented by national authorities, this could provide internal feedback to the organisations without concern about confidentiality issues and foreign powers or third-party interests.

### Supplementary Information

Below is the link to the electronic supplementary material.

Supplementary file1 (DOCX 26 KB)

## Data Availability

Due to the sensitivity of the results and potential third state interest, data are not openly available on individual organisation.

## Abbreviations

CBRNE: Chemical, Biological, Radiological, Nuclear, Explosive
MCI: Mass Casualty Incident
METHANE: M-Major Incident Declared, E-Exact location, T-Type of Incident H-Hazards, A-Access, N-Number of Casualties, E-Emergency Service
MI: Major Incident
NORCE: Norwegian research centre
PLIVO: Norwegian shorting for On-going life-threatening violence
