# Supplementary material for: Evaluation of prehospital preparedness for major incidents on a national level, with focus on mass casualty incidents
Source: Eur J Trauma Emerg Surg. 2023 Dec 20;50(3):945–57. doi: 10.1007/s00068-023-02386-7 (PMC11249512; doi:10.1007/s00068-023-02386-7)
Supplement: Supplementary file 1 — Supplementary file1 (DOCX 26 KB) [file 68_2023_2386_MOESM1_ESM.docx]

## Supplementary Information

Online Resource 1 Illustration of cooperation principle among Norwegian Rescue and Emergency Services

## Appendix 1.

Survey questions translated to English

1. Which Health Trust cover the geographic area you work in?

⃝ Finnmark Hospital Health Trust

⃝ University Hospital Northern-Norway

⃝ Nordland Hospital Health Trust

⃝ Helgeland Hospital Health Trust

⃝ Nord-Trøndelag Health Trust

⃝ St.Olavs Hospital Health Trust

⃝ Møre og Romsdal Health Trust

⃝ Førde Health Trust

⃝ Bergen Health Trust

⃝ Fonna Health Trust

⃝ Stavanger Health Trust

⃝ Akershus University Hospital Health Trust

⃝ Oslo University Hosital Health Trust

⃝ Innlandet Hospital Health Trust

⃝ Telemark Hospital Health Trust

⃝ Vestfold Hospital Health Trust

⃝ Østfold Hospital Health Trust

⃝ Sørlandet Hospital Health Trust

⃝Vestre Viken Health Trust

⃝ Did not know

⃝ Others (specify)

1. In which prehospital Rescue or Emergency Service do you work?

⃝ Ambulance Emergency Medical Service (vehicle/boat)

⃝ Local Rescue Central (Police)

⃝ Emergency Medical Call Central Regional

⃝ Primary Health Care Emergency Central for General Practitioners

⃝ Police Incident Commander

⃝ Rescue and Search helicopter Service

⃝ Rescue Incident Commander

⃝ Emergency Medical Call Central Local

⃝ Helicopter Emergency medical Service

⃝ Others (specify)

1. Specify your function and work

1. Which function have mandate to activate the hospitals MCI/MI plan in your region?

⃝ Emergency Medical Call Central Regional

⃝ Emergency Medical Call Central Local

⃝ Hospital Commando Group/Trauma centre

⃝ Early warning team at the hospital/ Trauma centre

⃝ Others (specify)

⃝ Did not know

1. Who is deciding the preparedness level at the hospitals within your Health Trusts

⃝ Health Trust Commando Group

⃝ Regional Health Authority Commando Group

⃝ Others (specify)

⃝ Did not know

1. Which function in Emergency Medical Call Central can activate contingency plan for MCI/MI when incoming call indicates a need?

⃝ Shift leader/Nurse 1

⃝ Others (specify)

⃝ Did not know

1. Is there a local contingency plan for MCI/MI

⃝ Yes

⃝ No

⃝ Did not know

1. Is there a regional contingency plan for MCI/MI

⃝ Yes

⃝ No

⃝ Did not know

1. If answered yes on question7 and 8. Is the local contingency plan adapted to the regional plan?

⃝ Yes

⃝ No

⃝ Did not know

1. Which of the following scenarios are described in the MCI/MI contingency plan in your organisation? (Scenarios described in plan, not necessary reflected in action cards) (Multiple answer question)

⃝ Fire

⃝ CBRNE

⃝ Epidemics

⃝ Hypothermia (drowning)

⃝ Changes in infrastructure

⃝ Inhalation injuries

⃝ MCI

⃝ Natural Disasters

⃝ Breakdown of network Phones/computers

⃝ Psychologic trauma

⃝ Terrorist Attack

⃝ Lack of water

⃝ Security threat

⃝ PLIVO

⃝ Others (specify)

⃝ Did not know

⃝ Contingency plan did not contain any specific scenarios

1. Who is responsible to revise the contingency plan for MCI/MI in your organisation?

⃝ Emergency Medical call, Central Doctor on call/ leader and Senior consultant for the Emergency Medical Section

⃝ Emergency Coordinator

⃝ Preparedness and disaster Command Group in the organisation

⃝ Others (specify)

⃝ Did not know

1. When was the contingency plan for MCI/MI last revised?

⃝ < 1 year

⃝ > 1 year

⃝ > 2 years

⃝ Yearly

⃝ Did not know.

1. Who is supposed to have knowledge about the contingency plan for MCI/MI in your organisation (single answer)

⃝ All new employees should read the contingency plan for MCI/MI

⃝ It is up to the individual employee to read contingency plan for MCI/MI when needed

⃝ We do not have any guidelines regarding knowledge of the contingency plan for MCI/MI

⃝ Others (specify)

⃝ Did not know

1. How is the knowledge among staff regarding contingency plan for MCI/MI controlled/followed-up? (Single answer)

⃝ Annual test

⃝ Web-based courses

⃝ Joint review

⃝ Trust (assume that all employees know their function)

⃝ No need to control/follow-up

⃝ Others (specify

⃝ Did not know

1. Has your organisation activated the MCI/MI contingency plan?

⃝ Yes, last 2 years

⃝ yes, 2-5 years ago

⃝ Never

⃝ Did not know

1. Do you utilize action cards in MCI events? (Single answer)

⃝ All functions have action card, known and used

⃝ All functions have action cards, but not used

⃝ Some functions have action card

⃝ No functions have action cards

⃝ Did not know

1. Which of the following medical services has action cards? (Multiple answers)

⃝ First ambulance on scene

⃝ Medical Incident commander

⃝ Medical leader Health

⃝ Transport responsible function

⃝ Sector Operational Leader

⃝ Hospital staff

⃝ General Practitioner on call

⃝ Primary Health Care Emergency Central

1. Describe the following statement regarding your Health trust:

During the Covid-19 pandemic the Commando Group in my Health trust have become clearer in the function as leading and coordination of hospital resources?

1    2   3   4    5   6    7   8   9 10

⃝ ⃝ ⃝ ⃝ ⃝ ⃝ ⃝ ⃝ ⃝ ⃝

Not at all   Too a large degree

1. Do your organisation use national guidelines SALT as primary triage tool for MCI/MI?

⃝ Yes

⃝ No

⃝ Did not know

⃝ Other primary triage tool (specify)

1. Do your organisation use national guidelines as secondary triage tool for MCI/MI?

⃝ Yes

⃝ No

⃝ Did not know

⃝ Other primary triage tool (specify)

1. Which function is fulfilling the role as Medical Incident Commander in an MCI/MI event? (Multiple answer)

⃝ First Ambulance on scene (most experienced ambulance worker)

⃝ First Ambulance on scene (other profession) (specify)

⃝ First Medical Doctor on scene

⃝ First Anaesthetic Doctor from helicopter on scene

⃝ Operational Leader Ambulance Service

⃝ Other (specify)

⃝⃝ Did not know

1. Which function is fulfilling the role as Medical Leader Health in an MCI/MI event? (Multiple answer)

⃝ First Ambulance on scene (most experienced ambulance worker)

⃝ First Ambulance on scene (other profession) (specify)

⃝ First Medical Doctor on scene

⃝ First arriving General Practitioner

⃝ First Anaesthetic Doctor from helicopter on scene

⃝ Operational Leader Ambulance Service

⃝ The person given the role by MIC in case of no Medical Doctor on scene

⃝ Other (specify)

⃝ Did not know

1. Which function is cooperating with PIC and RIC at the Coordination and Command site? (Multiple answer)

⃝ Medical Incident Commander

⃝ Medical Leader Health

⃝ Medical Incident Commander and Medical Leader Health

⃝ Operational Leader Ambulance Service

⃝ General Practitioner

⃝ Other (specify)

⃝ Did not know

1. Which function is responsible for medical decisions on scene? (Multiple answer)

⃝ Medical Incident Commander

⃝ Medical Leader Health

⃝ Medical Incident Commander and Medical Leader Health

⃝ Operational Leader Ambulance Service

⃝ General Practitioner

⃝ Other (specify)

⃝ Did not know

1. Which function is responsible for decision regarding patient distribution? (Multiple answer)

⃝ Medical Incident Commander

⃝ Medical Leader Health

⃝ Medical Incident Commander and Medical Leader Health

⃝ Operational Leader Ambulance Service

⃝ General Practitioner

⃝ Other (specify)

⃝ Did not know

1. Which function is responsible for arriving health resources to the scene of an MCI/MI event?

⃝ Medical Incident Commander

⃝ Medical Leader Health

⃝ Medical Incident Commander and Medical Leader Health

⃝ Operational Leader Ambulance Service

⃝ General Practitioner

⃝ Other (specify)

⃝ Did not know

1. Which medical competency has the function in question 26? (describe)

1. Did your organisation provide communication trainings for MCI/MI event?

⃝ Yes

⃝ No

⃝ Did not know

1. Has your organisation tested or planned for alternative communications methods in case of “Nødnett” breakdown?

⃝ Yes

⃝ No

⃝ Did not know

1. Are there guidelines in your organisation for the primary report form in case of MCI/MI event?

⃝ Yes, METHANE

⃝ Yes, others (specify)

⃝ No

⃝ Did not know

1. Are there local routines for structured follow-up after MCI/MI events?

⃝ Yes

⃝ No

⃝ Did not know

1. If yes in question 31. Describe how the follow-up is conducted

1. Is there a function in your organisation responsible for MCI/MI event evaluation and preparedness work?

⃝ Yes

⃝ No

⃝ Did not know

1. If yes in question 33. describe briefly

1. In case of on-going life-threatening violence who is responsible to make decision regarding performing first aid on scene? (Single answer)

⃝ Police Incident Commander

⃝ Rescue Incident Commander

⃝ Medical Incident Commander

⃝ Medical Leader Health

⃝ Joint decision between 2 or more Emergency Services

⃝ Other (specify)

⃝ Did not know

1. Do your contingency plan contain written information regarding how health personnel are supposed to act first arriving on scene in case of ongoing suspected terrorist attack? (Single answer)

⃝ Yes

⃝ No

⃝ Did not know

⃝ Not relevant

1. Is tactical first aid skill training (TEMS or similar) provided to one or more staff in your organisation? (Single answer)

⃝ Yes (specify number of staff and which training)

⃝ No

⃝ Did not know

⃝ Confidential

1. Is primary haemorrhagic skills training provided to one or more staff in your organisation? (Single answer)

⃝ Yes (specify number of staff and which training)

⃝ No

⃝ Did not know

⃝ Confidential

1. How many tourniquets carried in each unit (vehicle, boat, helicopter)? (Single answer)

⃝ >10

⃝ 8-9

⃝ 6-7

⃝ 4-5

⃝ 2-3

⃝ 1

⃝ Did not know

⃝ Confidential

1. When was the last time your organisation participated in Leadership and Coordination exercises with other Rescue and Emergency Services involving Hospitals conducted? (Single answer)

⃝ < 6 months

⃝ < 12 months

⃝ < 24 months

⃝ < 36 months

⃝ > 36 months

⃝ Never

⃝ Did not know

1. Do your organisation or region participate in MCI/MI events? (Multiple answer)

⃝ Internal Leadership and Coordination exercise

⃝ Simulation with marker or patient case

⃝ Combined Leadership and Coordination exercise with marker or Patient-case simulation

⃝ Leadership and Coordination exercise with other Prehospital Rescue and Emergency Services

⃝ Combined Leadership and Coordination exercise with marker or Patient-case simulation with other Prehospital Rescue and Emergency Services

⃝ Other exercise (Specify)

⃝ No exercises conducted

⃝ Did not know

1. When was the last internal Leadership and Coordination conducted, not involving other prehospital Emergency or rescue Services? (Single answer)

⃝ < 6 months

⃝ < 12 months

⃝ < 24 months

⃝ < 36 months

⃝ > 36 months

⃝ Never

⃝ Did not know

1. When did you last participate in Leadership and coordination exercise? (Single answer)

⃝ < 6 months

⃝ < 12 months

⃝ < 24 months

⃝ < 36 months

⃝ > 36 months

⃝ Never

⃝ Did not know

1. Which Rescue and Emergency Service usually participate in Leadership and coordination MCI/MI exercises? (Multiple answer)

⃝ EMCC

⃝ Police

⃝ Fire and Rescue Service

⃝ AEMS

⃝ PHC Call central

⃝ GP on call

⃝ Hospital/Hospital Command Group

⃝ HEMS

⃝ JRC

⃝ Other (specify)

⃝ Did not know

1. When was the last Patient-case simulation conducted in your organisation? (Single answer)

⃝ < 6 months

⃝ < 12 months

⃝ < 24 months

⃝ < 36 months

⃝ > 36 months

⃝ Never

⃝ Did not know

1. Which Rescue and Emergency Service usually participate in Patient-case simulations? (Multiple answer)

⃝ EMCC

⃝ Police

⃝ Fire and Rescue Service

⃝ AEMS

⃝ PHC GP office

⃝ GP on call

⃝ PHC Nurse at emergency medical call central

⃝ HEMS

⃝ Hospital Doctors

⃝ Hospital Nurses

⃝ Radiographers/bioengineers

⃝ Other (specify)

1. Which scenarios has been used in patient-case simulations? (Multiple answer)

⃝ Fire

⃝ Explosion

⃝ Transport accident

⃝ Chemical accident

⃝ Radio nuclear accident

⃝ PLIVO with firearms

⃝ PLIVO without firearms

⃝ Other (specify)

⃝ Did not know

1. How many local exercises/simulations with terrorist attack or disaster with multiple injured have been conducted last 5 years?

1. How many regional exercises/simulations with terrorist attack or disaster with multiple injured have been conducted last 5 years?

Importance grading of preparedness work for MCI/MI in your organisation or region

Scale 0-10 0= No importance, 10= Very important

1. Simulation and exercises in your organisation

0    1    2   3   4   5   6   7    8  9  10

⃝ ⃝ ⃝ ⃝ ⃝ ⃝ ⃝ ⃝ ⃝ ⃝ ⃝

1. Preparedness storage in your region

0    1    2   3   4   5   6   7    8  9  10

⃝ ⃝ ⃝ ⃝ ⃝ ⃝ ⃝ ⃝ ⃝ ⃝ ⃝

1. Improved communication

0    1    2   3   4   5   6   7    8   9  10

⃝ ⃝ ⃝ ⃝ ⃝ ⃝ ⃝ ⃝ ⃝ ⃝ ⃝

1. Clear leadership

0    1    2   3   4   5   6   7    8   9  10

⃝ ⃝ ⃝ ⃝ ⃝ ⃝ ⃝ ⃝ ⃝ ⃝ ⃝

1. Increased cooperation between different prehospital Rescue and Emergency Services in your region

0    1    2   3   4   5   6   7    8   9  10

⃝ ⃝ ⃝ ⃝ ⃝ ⃝ ⃝ ⃝ ⃝ ⃝ ⃝

1. Increased civilian and military cooperation in your region

0    1    2   3   4   5   6   7    8  9  10

⃝ ⃝ ⃝ ⃝ ⃝ ⃝ ⃝ ⃝ ⃝ ⃝ ⃝

1. Knowledge exchange with international prehospital Rescue and Emergency Services

0    1    2   3   4   5   6   7    8  9  10

⃝ ⃝ ⃝ ⃝ ⃝ ⃝ ⃝ ⃝ ⃝ ⃝

1. Research and professional development in MCI/MI preparedness in your organisation

0    1    2   3   4   5   6   7    8  9  10

⃝ ⃝ ⃝ ⃝ ⃝ ⃝ ⃝ ⃝ ⃝ ⃝ ⃝

1. Specify if there are other areas of importance in prehospital MCI/MI preparedness

1. Other comments
